# Association between dietary patterns and metabolic syndrome in individuals with normal weight: a cross-sectional study

**DOI:** 10.1186/s12937-015-0045-9

**Published:** 2015-05-30

**Authors:** Edyta Suliga, Dorota Kozieł, Elżbieta Cieśla, Stanisław Głuszek

**Affiliations:** 1 The Department for the Prevention of Alimentary Tract Diseases, The Institute of Nursing and Midwifery, Faculty of Health Sciences, Jan Kochanowski University, ul. IX Wieków Kielc 19, 25-317 Kielce, Poland; 2 The Department of Surgery and Surgical Nursing with the Scientific Research Laboratory, The Institute of Nursing and Midwifery, Faculty of Health Sciences, Jan Kochanowski University, ul. IX Wieków Kielc 19, 25-317 Kielce, Poland; 3 Department of Developmental Age Research, Institute of Public Health, Faculty of Health Sciences, Jan Kochanowski University, ul. IX Wieków Kielc 19, 25-317 Kielce, Poland

**Keywords:** Dietary patterns, Metabolic syndrome, Body mass index

## Abstract

**Background:**

The results of several papers have confirmed the existence of correlations between an unhealthy diet and the presence of metabolic syndrome. However, relationships between eating habits and metabolic obesity with normal weight have not yet been sufficiently studied. The aim of the study is to determine which dietary patterns are present in individuals with a normal BMI and to find out whether those patterns were connected with the risk of metabolic syndrome and its features.

**Methods:**

A cross-sectional study was carried out in a group of 2479 subjects with a normal weight (BMI = 18.5–24.9 kg/m^2^), aged between 37–66. The study included the evaluation of eating habits, anthropometric measurements, blood pressure tests and the analysis of the collected fasting-blood samples, on the basis of which cholesterol, triglycerides and glucose levels were determined. Dietary patterns were determined by means of factor analysis.

**Results:**

In the group of individuals with a normal BMI, four dietary patterns were distinguished: “healthy”, “fat, meat and alcohol”, “prudent” and “coca cola, hard cheese and French fries”. After controlling for potential confounders, subjects in the highest tertile of prudent dietary pattern scores had a lower odds ratio for the metabolic obesity normal weight) (odds ratio: 0.69; 95 % CI: 0.53-0.89; *p* < 0.01) and low HDL cholesterol (odds ratio: 0.77; 95 % CI: 0.59-0.99; *p* < 0.05), in comparison to those from the lowest tertile, whereas the individuals in the second tertile had a higher odds ratio for the increased blood glucose concentration than those in the lowest tertile (odds ratio: 0.74; 95 % CI: 0.57-0.96; *p* < 0.05).

**Conclusion:**

A dietary pattern characterized by a high consumption of fish and whole grains, and a low consumption of refined grains, sugar, sweets and cold cured meat, is connected with lower risk of metabolic obesity normal weight as well as with the lower risk of low HDL cholesterol concentration and increased glucose concentration.

## Introduction

Metabolic syndrome most often co-occurs with obesity, however, in some individuals with normal body weight, the presence of metabolic disorders can be diagnosed similar to those which are characteristic for obese people. These persons were named metabolically obese normal weight (MONW) [1]. Metabolic obesity normal weight is diagnosed in 14.7 % - 59 % adults in different populations [2–4]. Significant differences concerning the frequency of the occurrence of this problem result from the diversified age and ethnic origin of the subjects, as well as from the varied diagnosing criteria [3, 4].

The results of several studies have confirmed the dependencies between an unhealthy diet and the occurrence of the metabolic syndrome features, whereas “healthy” dietary patterns decreased this risk [5–8]. In the etiology of metabolic syndrome with normal weight, the significance of an improperly balanced diet is underlined [3, 9, 10]. The analyses used in the food epidemiology of the relationships between particular nutrients with the presence of the metabolic syndrome features, often indicated the lack of such correlations. It is difficult to detect an effect caused by single nutrients. Thus, the analysis of the dietary patterns seems to be useful in the assessment of the dependencies between eating habits and the occurrence of chronic diseases. They reflect eating habits of individuals in populations more comprehensively, because what people eat is not nutrients, but products and meals consisting of diversified food, which represent the total effect of individual nutrients and the interactions between them. They are also more useful in working on dietary recommendations.

The dietary patterns are most often determined by means of factor or cluster analysis, and in the case of these two methods, they are connected with the risk of the presence of chronic diseases. However, Newby et al. showed that a person’s dietary patterns would be best represented by using factor analysis [11]. The studies also confirmed reproducibility and validity of the dietary patterns determined by factor analysis in various populations [12, 13]. The aim of the study is to determine which dietary patterns occur in individuals with a normal BMI and to check if these patterns are connected with the risk of the presence of metabolic syndrome and its features.

## Material and methods

The research material was collected in the framework of the PONS project (Polish-Norwegian Study) [4, 14]. The study was approved by the Ethics Committee within the Cancer Centre and Institute of Oncology in Warsaw, Poland. The study was carried out from September 2010 to October 2012. It was an open-ended study. All men and women aged between 45–64, permanently residing in the Kielecki region in Poland, were invited to take part in the study. Voluntary participation rate in this age group was 12 %. A small number of older people volunteered to take part in the study (65–66 years), and younger (37–44), in comparison with the previously intended age group. They comprised 0.66 % of all participants and were included in the analysis. Altogether 13172 people were subjected to the study (4447 men and 8725 women) aged 37 – 66. In 65 subjects (0.49 %), due to the lack of information concerning their height or body mass, it was not possible to calculate a BMI, 73.69 % were obese and overweight people, 0.44 % were underweight. 3342 participants with a normal BMI were qualified for further analysis (18.5 – 24.9 kg/m^2^). Following this, the participants with a history of cardiovascular system disease, strokes, diabetes or cancer were excluded from the analysis – due to possible changes in their diet resulting from disease (*N* = 193). The participants lacking data concerning the consumption of particular products were also excluded (*N* = 670). The dietary patterns were established on the basis of factor analysis carried out in the remaining 2479 individuals. Participants with a normal BMI, in comparison with all subjects, were significantly more frequently women, younger people, living in cities, having higher education, more physically active and smoking (Table 1). The differences are in accordance with the results of studies stating that overweight and obesity are less common in socio-economically privileged groups, especially in women [15, 16]. The participants with a normal BMI, not having complete data, were not significantly different than those included in the analysis from the point of view of sex and place of residence, however, they were older, less physically active, they had higher education less frequently, and were smokers more often. The studies included a questionnaire interview, anthropometric measurements, blood pressure measurements and analyses of collected fasting-blood samples on the basis of which the concentration of cholesterol, triglycerides and glucose was determined.

**Table 1:** The characteristics of the study participants (%, 95 % CI)

The study participants	Socio-demographic variables and life style					
	Women	Age 52–66 years	Rural environm.	University education	Smokers	Physical activity*
Total population N = 13172	66.2	75.2	38.2	29.5	20.0	17.3 ± 11.1
	65.4-67.0	74.3-79.9	37.4-39.0	28.7-30.3	19.3-20.7	17.1-17.5
Normal BMI – missing data N = 670	77.0^a^	74.2^a^	33.6^a^	29.7^a^	30.4^a^	17.0 ± 10.7^a^
	74.1-79.8	71.2-77.1	27.5-33.8	26.6-32.8	27.3-33.5	10.2-11.2
The subject group N = 2479	76.2	65.8^b^	32.1	37.9^b^	28.2^b^	17.8 ± 11.0^b^
	74.8-77.7	64.2-67.4	30.6-33.8	36.3-39.6	26.7-29.8	17.5-18.2

*X ± SD (MET h/week); ^a^statistically significant difference between the subject group and a total population *p* < 0.05; ^b^statistically significant difference between the group with a normal BMI and missing data and the subject group, *p* < 0.05

### Anthropometric measurements

The measurements of body weight were done by means of an electronic weight Tanita SC 240 MA, with an accuracy of 0.1 kg. Body height measurements were done by means of the weight’s stadiometer, with an accuracy of 0.1 cm. The waist circumference was measured halfway between the lower rib edge and the upper iliac crest by means of a metric measure with an accuracy of 0.1 cm.

### Assessment of blood pressure and biomarkers

Blood pressure was measured with the use of the blood pressure monitor Omron, model M3 Intellisense. The test was carried out on the artery of the right upper limb, when seated. In the study, the average of the two measurements was analysed. The glucose concentration in the blood serum was determined by means of the enzyme method with hexokinase, the concentration of triglycerides – by means of the phosphogliceride oxidase-peroxidase method. The concentration of total cholesterol was determined by means of the enzyme method with esterase and cholesterol oxidase, and HDL cholesterol with the use of the colorimetric non-precipitation method.

### Assessment of other variables

The confounders included the variables which affect both the dietary patterns as well as the features of metabolic syndrome such as age, level of education, place of residence, smoking cigarettes and physical activity. Two 15-year age brackets were distinguished: 37–51 and 52–66, four education categories: primary, vocational, secondary and university as well as two categories for the place of residence: urban and rural. The physical activity was evaluated with the use of the International Physical Activity Questionnaire (IPAQ). Total physical activity was calculated expressed as metabolic equivalent hours per week (MET-h/wk). The respondents who smoked cigarettes on a daily basis during the study were classified as current smokers, and those who had not smoked for longer than 6 months – as former smokers, and the rest was regarded as nonsmokers.

### Food consumption analysis

The data concerning food consumption were collected by means of the Food Frequency Questionnaire (FFQ). FFQ consisted of a list of products of standard size portions. The participants of the study were asked about the frequency of consumption of certain portions of each product during the last year. The frequencies of consumption were classified as follows: less frequently than once a month or not at all, 1–3 times a month, once a week, 2–4 times a week, 5–6 times a week, once a day, 2–3 times a day, 4–5 times a day, 6 times a day or more, I don’t know, I refuse to answer the question. In order to achieve the dietary patterns, food items and beverages from the questionnaire were combined in 31 food groups. The method of aggregating the products is shown in Table 2. The basis for placing a given product in a group was a similarity of nutritional value. Some food items were analysed separately because the profile of their ingredients was unique (e.g. eggs, margarine) or their consumption could reflect a distinct dietary pattern (e.g. bigos – a traditional Polish dish made of sauerkraut, sausages and meat). PONS FFQ was constructed based on a previously developed and validated FFQ for the Poland branch of the PURE study and was characterized by good validity and reproducibility in relation to the referential method [17].

**Table 2:** Food grouping used in the dietary pattern analyses

Food groups	Food items
Refined grains	White bread, pasta
Whole grains	Dark bread, corn flakes, groats
Vegetables	Red beetroots, raw white cabbage, broccoli, carrots, garlic, onions, tomatoes, green leafy vegetables
Fruit	Apples, bananas, oranges, seasonal fruits (berries)
Nuts	Nuts
Whole milk	Whole milk
Low-fat milk	Low-fat milk
Cottage cheese	Cottage cheese
Yogurt	Yogurt
Hard cheese	Hard cheese
Fish	Fish
Chicken	Chicken
Eggs	Eggs
Red meat	Pork, beef, liver
Cold cured meat	Bacon, ham, sausages, frankfurters
Lard	Lard
Butter	Butter
Vegetable oils	Rapeseed, soya, sunflower oil, olive oil, and other oils
Margarine	Margarine
Mayonnaise	Mayonnaise
French-fries	French-fries
Boiled potatoes	Boiled potatoes
Bigos	Bigos (sauerkraut, sausage and meat stew)
Fried foods	Fried foods
Sugar and sweets	Sugar, chocolate and chocolate-products, candies, cakes, cookies
Fruit juice	Fruit juice
Diet carbonated soft drinks	Diet carbonated soft drinks
Coca cola	Coca cola or other beverages of this type
Alcoholic beverages	Beer, fruit wine, grape wine, vodka
Coffee	Coffee
Tea	Tea

### Definition of MONW

The occurrence of metabolic syndrome was defined based on the recommendations of the International Diabetes Federation Task Force on Epidemiology and Prevention (joint interim statement in 2009) [18]. Metabolic obesity with normal weight (MONW) was defined as the presence of at least three of the 5 features of metabolic syndrome and a normal body weight (BMI = 18.5-24.9 kg/m^2^).

### Statistical analysis

The dietary patterns were distinguished with the use of factor analysis by principal component analysis. The answers concerning the frequency of consumption of particular items from the questionnaire were calculated into daily doses of consumption and then standardized by z-score. Barlett’s test of sphericity was conducted (*p* < 0.0000), KMO coefficient was calculated (Kaiser-Meyer-Olkin) (>0.7) and for each variable, MSA (measure sampling adequacy) was counted. The applied procedure allowed us to eliminate the products which did not show any connection with other products or were weakly correlated with them. From further analysis the following products were excluded: whole milk, butter, chicken, nuts, dietetic sparkling drink, coffee and tea. The analysis of the principal components with varimax rotation allowed us to minimize the number of variables which had high loads on each factor. The first factor extracted is the one that accounts for the maximum possible variance in the dataset. The second component, independent of the first, will be the one that explains the largest possible share of the remaining variance and so on, without the components being correlated with each other. The number of factors was determined in accordance with the Kaiser criterium (>1). Only these factors were included which were responsible for variances not smaller in number than the original variables. Additionally, the number of the accepted factors was included by the scree plot test. Each factor consisted of subsets of food items subject to analysis, whose absolute factor loading was greater than or equal 0.3. The factor analysis carried out led to distinguishing 4 factors. The contribution of specific factors to the explanation of total variance was as follows: the first factor explained: 12.74 %, the second factor: 9.69 %, the third factor 5.50 %, the fourth factor: 5.02 %. The selected factors altogether allowed for 32.95 % of the total variance. On the basis of own scores of the consumption of a given product or groups of products and standardized weights from the factor analysis for every subject factor, values were calculated for each dietary pattern. In the range of each pattern the division of subjects into tertile groups was carried out and the characteristic of the subjects in tertile groups was made, in the range of dietary patterns. In reference to the socio-demographic factors (age, sex, place of residence, level of education), smoking, and also for the features of metabolic syndrome, in particular dietary patterns the structure indicator was tested with the use of non-parametric chi-square test. To evaluate physical activity one factor variance analysis ANOVA and post-hoc Bonferroni test were used. In order to assess the risk (OR) of the MONW occurrence as well as improper values of 5 features of metabolic syndrome in the distinguished dietary patterns logistic regression was applied. The values of the first, lowest tertile (T1) were accepted as a reference level for each dietary pattern. In the first model crude data were considered, whereas in the second model the risk of the occurrence of improper values of MONW features was adjusted to the age (37–51, 52–66), sex (women, men), place of residence (city, country), education (higher, secondary and lower), smoking (never, past, or current) and total physical activity (MET-h/wk). All p-values presented are 2-tailed; *p* < 0.05 was considered significant. In order to compare the selected research sample with the entire population as well as people with incomplete data, in reference to quality variables test chi-square was used, whereas for physical activity T-Student test was applied. The statistical analysis was carried out with the use of Statistica program, version 10.0.

## Results

The factor analysis allowed us to distinguish four dietary patterns in the group of participants with a normal BMI: pattern I – healthy, most strongly correlated with the consumption of fruit and vegetables, low-fat milk and dietary products, as well as whole grains food, pattern II - fat, meat and alcohol – correlated with the consumption of lard, red meat, cold cured meat, eggs, fried dishes, vegetable oils, mayonnaise and alcoholic drinks, pattern III – prudent – additionally correlated with the consumption of fish and whole grains products, whereas negatively correlated with the consumption of refined grains food, sugar and sweets, cold cured meats and boiled potatoes and pattern IV – coca cola, hard cheese and French fries – positively correlated with the consumption of these three products. The factor-loading matrixes for these dietary patterns are shown in Table 3.

**Table 3:** Factor-loading matrix for major dietary patterns*

Food groups	Factor I	Factor II	Factor III	Factor IV
Alcoholic beverages	-	0.302	-	-
Low-fat milk	0.363	-	-	-
Cottage cheese	0.538	-	-	-
Yogurt	0.449	-	-	-
Hard cheese	-	-	-	0.499
Fish	-	-	0.343	-
Eggs	-	0.593	-	-
Red meat	-	0.452	-	-
Cold cured meat	-	0.333	−0.416	-
Lard	-	0.441	-	-
Fried foods	-	0.491	-	-
Vegetable oils	-	0.384	-	-
Mayonnaise	-	0.395	-	-
French-fries	-	-	-	0.502
Coca cola	-	-	-	0.579
Fruit juice	-	-	-	-
Fruit	0.662	-	-	-
Vegetables	0.626	-	-	-
Boiled potatoes	-	-	−0.624	-
Whole grains	0.329	-	0.583	-
Refined grains	-	-	−0.702	-
Sugar and sweets	-	-	−0.551	-
Percentage of variance explained (%)	12.742	9.693	5.497	5.029

*Values < 0.30 were excluded for simplicity

Characteristics of the study participants across the tertile categories of the dietary pattern scores are shown in Table 4. In comparison with the subjects from the first tertile (T1) of a healthy pattern, in the second and third tertile (T2 and T3), women were found significantly more often, as well as the participants with higher level of education, to be living in the city, non-smokers and more physically active. Similarly, the participants in T2 and T3 of the prudent pattern, in comparison with those in T1, were more often women, people with a higher education, living in cities and non-smokers. T3 included the highest number of former smokers compared to T1 and T2. In this pattern there were no significant differences of physical activity between the participants in other tertiles. The participants in T2 and T3 of the pattern fat, meat, alcohol in comparison with those in T1, were more often men, smokers, individuals living in cities and of higher total physical activity. In case of the pattern of coca cola, hard cheese and French fries, the subjects in T2 and T3 were younger, more physically active and more frequently resided in the country compared to those in T1. Moreover, the participants in T3 of this pattern were more often smokers.

**Table 4:** The characteristic of the study participants in the tertile categories of the dietary patterns in respect of demographic, social aspect and lifestyle (%, 95 % CI)

DP	T	Demographic and social variables and lifestyle					
		Women	Age 52–66 years	Rural environm.	University education	Smokers	Physical activity*
I	T1	64.0	61.7	35.4	34.1	34.9	15.6 ± 10.4
		60.6-67.2	58.3-65.0	32.1-38.8	30.8-37.4	31.7-38.3	14.9-16.3
	T2	78.0	63.2	32.9	41.6	25.3	18.1 ± 10.6
		75.0-80.8	59.9-66.5	29.7-36.2	38.2-45.0	22.3-28.4	17.4-18.8
	T3	85.97	63.0	26.6	45.9	22.0	20.5 ± 11.5
		83.4-88.3	59.6-66.3	23.6-29.8	42.5-49.4	19.3-25.0	19.7-21.3
	p	0.0000	ns	0.0004	0.0000	0.0003	0.0000
II	T1	84.3	65.0	33.4	38.7	23.9	17.2 ± 10.5
		81.6-86.7	61.7-68.3	30.2-36.8	35.4-42.2	21.0-26.9	16.5-17.9
	T2	78.7	62.0	33.41	40.8	27.2	18.2 ± 10.6
		75.7-81.4	58.6-65.3	30.2-36.8	37.4-44.2	24.2-30.4	17.4-18.9
	T3	65.1	60.9	28.1	42.1	31.1	18.9 ± 11.9
		61.7-68.3	57.5-64.3	25.0-31.3	38.7-45.5	27.9-34.4	18.1-19.7
	p	0.0000	ns	0.0256	ns	0.0001	0.0063
III	T1	67.1	61.0	44.3	25.9	38.1	18.0 ± 11.1
		63.8-70.3	57.6-64.4	40.9-47.8	23.0-29.0	34.8-41.6	17.3-18.8
	T2	78.1	63.6	30.7	40.4	25.5	17.9 ± 10.9
		75.1-80.9	60.2-66.9	27.6-34.0	37.0-43.8	22.6-28.6	17.2-18.7
	T3	82.81	63.3	19.9	55.3	18.6	18.3 ± 11.1
		80.1-85.3	59.9-66.6	17.2-22.7	51.9-58.8	16.0-21.4	17.5-19.0
	p	0.0000	ns	0.0000	0.0000	0.0000	ns
IV	T1	78.2	68.6	28.7	39.5	27.4	17.1 ± 10.6
		75.2-81.0	65.4-71.8	25.6-31.9	36.1-42.9	24.4-30.5	16.4-17.9
	T2	75.9	65.8	30.5	41.5	25.2	18.2 ± 10.8
		72.9-78.8	62.4-69.0	27.4-33.7	38.1-44.9	22.2-28.3	17.4-18.9
	T3	73.9	53.5	35.7	40.7	29.7	18.9 ± 11.6
		70.7-76.8	50.0-57.0	32.4-39.1	37.3-44.1	26.6-32.9	18.1-19.7
	p	ns	0.0000	0.0061	ns	ns	0.0046

*T* tertile, *ns* non significant

*X ± SD (MET h/week)

The subjects in T2 and T3 of the health and prudent patterns, compared to those in T1, had increased glucose and triglycerides concentrations and less frequently a low concentration of HDL cholesterol (Table 5). Only the percentage of individuals with abdominal obesity, in the case of these two patterns, was higher in T2 than in T1 and T3. The analysis of the waist circumference, considered as a continuous variable, showed however, that the higher the score of the healthy and prudent patterns, the lower was the average waist circumference of the subjects (Table 6). The individuals in T3 of the pattern fat, meat, alcohol were characterized by a higher glucose and triglycerides concentration, compared with those from T2 and T1 (Table 5). They were also characterized by higher average waist circumferences (Table 6), despite the fact that no significant differences were found concerning the occurrence of abdominal obesity. In the pattern “coca cola, hard cheese and French fries” there were no significant differences in the occurrence of the metabolic syndrome features through tertiles. MONW occurred less frequently in individuals in T3 of the healthy and prudent patterns, compared to those in T2 and T1. No significant differences were observed between the frequency of MONW in the subjects from other tertiles of the pattern “fat, meat and alcohol” as well as the pattern “coca cola, hard cheese and French fries”.

**Table 5:** The characteristic of the study participants in the tertile categories of the dietary patterns in respect to the metabolic syndrome features occurrence (% and 95 % CI)

Dietary patterns	T	Abnormal values of metabolic syndrome features					MONW
		Waist circumf.	Fasting glucose	Triglycerides	HDL cholest.	Blood pressure	
I	T1	24.6	22.1	29.7	24.4	66.2	25.1
		21.7-27.7	19.3-25.1	26.7-33.0	21.5-27.4	62.8-69.4	22.2-28.2
	T2	30.4	17.8	26.0	22.49	66.5	23.8
		27.2-33.6	15.2-20.6	23.0-29.0	19.7-25.5	63.2-69.7	20.9-26.9
	T3	26.8	15.4	20.44	19.2	63.4	18.5
		23.9-30.0	13.0-18.0	17.7-23.4	16.6-22.1	60.0-66.7	15.9-21.3
	p	0.0304	0.0017	0.0001	0.0386	ns	0.0030
II	T1	28.6	16.2	23.1	23.2	63.1	21.4
		25.5-31.8	13.8-18.9	20.3-26.2	20.4-26.3	59.1-66.4	18.7-24.4
	T2	28.4	16.4	24.3	20.9	64.6	21.3
		25.3-31.6	14.0-19.1	21.4-27.3	25.5-32.1	61.3-67.9	18.5-24.2
	T3	24.9	22.6	28.7	21.9	68.4	24.7
		21.9-27.9	19.7-25.6	25.7-32.0	19.2-24.9	65.1-71.5	21.8-27-8
	p	ns	0.0008	0.0219	ns	ns	ns
III	T1	28.3	22.6	30.5	26.5	65.5	27.9
		25.3-31.5	19.8-25.7	27.4-33.8	23.5-29.7	62.1-68.7	24.8-31.0
	T2	29.3	15.7	23.6	20.4	66.0	21.4
		26.2-32.5	13.3-18.4	20.7-26.6	17.7-23.3	62.7-69.3	18.6-24.3
	T3	24.2	16.8	22.0	19.1	64.5	18.2
		21.3-27.3	14.3-19.6	19.3-25.0	16.5-22.0	61.2-67.8	15.6-21.0
	p	0.0494	0.0005	0.0001	0.0005	ns	0.0000
IV	T1	26.4	18.8	27.0	23.2	66.5	24.2
		23.4-29.5	16.2-21.6	24.0-30.2	20.4-26.3	63.1-69.7	21.3-27.3
	T2	27.6	19.2	23.9	20.6	65.2	22.6
		24.6-30.8	16.6-22.1	21.1-27.0	17.9-23.5	61.8-68.4	19.8-25.6
	T3	27.9	17.2	25.2	22.3	64.4	20.6
		24.8-31.0	14.7-19.9	22.3-28.3	19.5-25.3	61.0-67.7	17.9-23.5
	p	ns	ns	ns	ns	ns	ns

*T* tertile, *ns* non significant

**Table 6:** Waist circumferences in tertile categories of the dietary patterns (X ± SD; 95 % CI)

Tertile	Dietary patterns			
	Healthy	Fat, meat and alcohol	Prudent	Coca cola, hard cheese and French-fries
T1	80.9 ± 7.7	78.5 ± 7.2	80.5 ± 7.6	79.0 ± 7.6
	80.3-81.4	78.0-79.0	80.0-81.0	78.5-79.5
T2	79.4 ± 7.6	79.2 ± 7.4	79.4 ± 7.9	79.1 ± 7.6
	78.9-79.9	78.7-79.9	78.8-79.9	78.6-79.6
T3	77.5 ± 7.0	80.0 ± 7.9	77.9 ± 6.9	79.6 ± 7.2
	77.0-78.0	79.5-80.6	77.4-78.3	79.1-80.1
p	0.0000	0.0001	0.0000	ns

In the model based on the crude data analysis, the risk of MONW, as well its three features, i.e. an increased glucose and triglycerides concentration and low concentration of HDL cholesterol, was significantly lower in the participants in T2 and T3 of the healthy and prudent patterns, compared to those from T1 (Table 7). A greater risk of the increased glucose and triglycerides concentration was present in individuals in T3, in comparison with the subjects in T1 of the fat, meat, alcohol pattern. The score of the coca cola, chard cheese and French fries pattern, was not significantly correlated with the risk of MONW and its features. In the model adjusted to sex, age, place of residence, education, overall physical activity and smoking, only the score of the prudent pattern was significantly correlated with the development of MONW and some of its features, i.e. a low concentration of HDL cholesterol and increased glucose concentration (Table 8). A higher MONW risk and low concentration of HDL cholesterol were found in participants from T3 in this pattern, compared to those from T1, whereas in the subjects from T2, the risk of an increased glucose concentration was significantly higher than in the participants in T1. It was also discovered that in participants in T2 of the healthy pattern, the risk of abdominal obesity was significantly higher than in those from T1.

**Table 7:** Risk (OR) of MONW and its features development in tertile categories of the dietary patterns (Model I – crude data) (OR; 95 % CI)

		Abnormal values of metabolic syndrome features					
Dietary patterns	T	Waist circumf.	Fasting glucose	Triglycerides	HDL cholest.	Blood pressure	MONW
I	T1	1.0	1.0	1.0	1.0	1.0	1.0
Healthy	T2	1.13**	0.76*	0.83	0.90	0.99	0.93
		0.93-1.39	0.60-0.98	0.66-1.03	0.72-1.13	0.81-1.21	0.74-1.16
	T3	0.89	0.64**	0.61***	0.73*	1.13	0.68**
		0.71-1.11	0.50-0.82	0.48-0.76	0.58-0.94	0.93-1.39	0.53-0.86
II	T1	1.0	1.0	1.0	1.0	1.0	1.0
Fat, meat and alcohol	T2	1.01	1.01	1.06	0.86	0.94	0.98
		0.81-1.25	0.78-1.31	0.85-1.33	0.68-1.08	0.77-1.15	0.77-1.24
	T3	1,21	1.50**	1.34**	0.94	0.79	1.22
		0.97-1.51	1.18-1.93	1.08-1.68	0.74-1.18	0.64-0.97	0.97-1.53
III	T1	1.0	1.0	1.0	1.0	1.0	1.0
Prudent	T2	0.95	0.64***	0.70**	0.71**	0.98	0.71**
		0.77-1.18	0.50-0.82	0.56-0.87	0.57-0.89	0.80-1.20	0.56-0.88
	T3	1.24	0.69**	0.64***	0.65***	1.04	0.57***
		0.99-1.54	0.54-0.88	0.52-0.80	0.52-0.83	0.85-1.28	0.45-0.73
IV	T1	1.0	1.0	1.0	1.0	1.0	1.0
Coca cola, hard cheese, French-fries	T2	0.94	1.03	0.85	0.85	1.06	0.91
		0.76-1.17	0.81-1.32	0.68-1.06	0.68-1.08	0.86-1.30	0.73-1.15
	T3	0.93	0.90	0.91	0.95	1.09	0.81
		0.75-1.15	0.70-1.16	0.73-1.13	0.71-1.19	0.89-1.34	0.64-1.02

*T* tertile

*p < 0.05; **p < 0.01; ***p < 0.001

**Table 8:** Risk (OR) of MONW and its features development in tertile categories of the dietary patterns (Model II – adjusted for potential confounders) (OR; 95 % CI)

		Abnormal values of metabolic syndrome features					
Dietary patterns	T	Waist circumf.	Fasting glucose	Triglycerides	HDL cholest.	Blood pressure	MONW
I	T1	1.0	1.0	1.0	1.0	1.0	1.0
Healthy	T2	0.81*	0.91	0.99	1.03	0.86	1.11
		0.67-0.99	0.71-1.18	0.80-1.23	0.81-1.31	0.70-1.07	0.88-1.40
	T3	1.00	0.84	0.79	0.90	0.92	0.87
		0.90-1.10	0.64-1.10	0.62-1.01	0.70-1.15	0.74-1.15	0.68-1.13
II	T1	1.0	1.0	1.0	1.0	1.0	1.0
Fat, meat and alcohol	T2	0.92	0.95	1.02	0.82	0.98	0.94
		0.74-1.15	0.73-1.24	0.80-1.29	0.65-1.04	0.79-1.20	0.74-1.20
	T3	0.93	1.25	1.13	0.79	0.93	1.04
		0.74-1.17	0.96-1.62	0.90-1.44	0.62-1.01	0.75-1.15	0.82-1.33
III	T1	1.0	1.0	1.0	1.0	1.0	1.0
Prudent	T2	0.98	0.74*	0.81	0.79	0.89	0.79
		0.78-1.22	0.57-0.96	0.64-1.02	0.62-1,00	0.71-1.10	0.62-1.00
	T3	1.22	0.88	0.80	0.77*	0.92	0.69**
		0.96-1.55	0.67-1.16	0.63-1.02	0.59-0.99	0.73-1.15	0.53-0.89
IV	T1	1.0	1.0	1.0	1.0	1.0	1.0
Coca cola, hard cheese, French-fries	T2	0.90	1.03	0.84	0.85	1.07	0.91
		0.72-1.13	0.80-1.33	0.67-1.06	0.67-1.07	0.86-1.32	0.72-1.16
	T3	0.88	0.91	0.90	0.94	1.04	0.82
		0.70-1.10	0.70-1.19	0.72-1.14	0.74-1.19	0.84-1.29	0.64-1.04

*T* tertile

*p < 0.05; **p < 0.01

## Discussion

In the group of participants having a normal BMI, 4 dietary patterns were identified: “healthy”, “fat, meat and alcohol”, “prudent” and “coca cola, hard cheese and French fries”. Significant correlations were found between the score of the dietary pattern and demographic and social variables, as well as a lifestyle. The subjects with a high score of the healthy and prudent pattern more often included women, nonsmokers, people with a university level of education, living in cities, and in case of the healthy pattern – also more physically active. The research results by other authors also confirm the existence of similar correlations. Fonseca et al. stated that the “healthy” dietary pattern was more frequent in the oldest women age group, in the pattern “red meat and alcohol” women with the lowest level of education were the most numerous, and in men – the biggest number of the youngest groups representatives [19]. Desmukh-Taskar et al. found that women consumed more portions from the “prudent” dietary pattern than men, and the individuals with a higher SES and a higher level of education, consumed more of them than subjects with a lower level of education and a lower social status [20]. The correlation between a higher education and higher SES, and the “prudent” dietary pattern results from more extensive knowledge, as well as health awareness, and also from the fact that being a member of a higher social group obliges to the dietary habits acknowledged and accepted by this group [20, 21]. Among people of lower SES, the important factors influencing the choice of food items include also a higher cost of healthy food or a poor access to health food [21, 22]. The results of the research carried out confirmed the tendency noticed by several authors of the coexistence of healthy eating habits with other health promoting elements of a lifestyle in individual people. Desmukh-Taskar et al. found out that current smokers consumed more portions of the “Western” dietary pattern, and non-smokers – more portions from the “prudent” pattern; the individuals of the highest physical activity consumed fewer portions from the “Western” dietary pattern than those less active [20]. Fonseca et al. discovered that the “healthy” dietary pattern both in men and women was connected with the lowest number of current smokers and people who drink alcohol, and also with the highest proportion of those who are physically active [19]. In the pattern “red meat and alcohol” there was a lowest percentage of physically active individuals, and among men – there was the highest number of current smokers.

The research conducted in recent years has shown that the genes expression profiles are different depending on dietary patterns, which may be a factor modulating the risk of chronic diseases development [23]. A vegetarian dietary pattern was connected with a more advantageous metabolic profile [24]. The risk of metabolic syndrome development was also lower in case of a greater consumption of dairy products [25]. However, the meat dietary pattern was strongly correlated with the occurrence of the syndrome [26]. Philips et al. concluded that individuals with MONW, in comparison with healthy people, were characterized by a lower consumption of vegetables, fruit and dairy products and a worse diet quality defined by the DASH (Dietary Approaches to Stop Hypertension score) [3]. A significant role of a lower level of dietary restraint in individuals with MONW was indicated [27]. The conducted study revealed that a lower risk of metabolic syndrome and its features, such as an increased glucose and triglycerides concentration and a low concentration of HDL cholesterol characterized the participants with a high score in the patterns “healthy” and “prudent”, whereas a higher risk of an increased glucose and triglycerides concentration was connected with the highest score in the pattern “fat, meat, alcohol”. After introducing control over confounding factors, a significantly lower risk of the MONW development and its features was found only in the participants with a high score in the “prudent” pattern, positively correlated with the fish and full grains food consumption. This pattern was also characterized by limiting the consumption of products that may be related to a disadvantageous metabolic profile, such as refined flower products, sugar, sweets and cold cured meat. The achieved results are in accordance with the results obtained by other authors who concluded that lowering the risk of metabolic syndrome and its features may be connected with the consumption of fish [28, 29]. Fish contribute to the improvement of the metabolic profile mainly due to a high content of polyunsaturated fatty acids n-3 [30]. Esmaillzadeh et al. showed that the consumption of full grain products was negatively, and the consumption of refined grain products positively correlated with the risk of the metabolic syndrome development [31]. Whole grains products contribute to the improvement of the metabolic profile due to the content of such components as: dietary fiber, inulin, beta-glucan, resistant starch, phenolics, tocotrienols, tocopherols, and carotenoids [32]. Hyun et al. having analysed eating habits of women with MONW, confirmed that they consumed more animal fats, and less full grain products and vegetables compared to the control group [9]. The risk of MONW was also increased by the consumption of carbohydrates, especially in the form of snacks [10]. After introducing control over the confounding variables, the other dietary patterns were no longer significantly correlated with the risk of MONW. The lack of such correlations was also noted down in the papers of other authors [19, 33]. It may result from the fact that the other groups of subjects were not concentrated in the way that their lifestyle, eating habits and socio-demographic features were significantly connected with the risk of the metabolic syndrome development.

### Study limitations

The main limitation of this paper is that the study was conducted “a posteriori”, therefore any changes of the dietary pattern which could take place in case of the subjects, can confound the possibility of a precise explanation of the causal link between the nutrition and the occurrence of MONW. Moreover, a lower number of men who volunteered to participate in the study, may cause that their dietary patterns were not fully representative for the whole population.

## Conclusions

The dietary pattern characterized by a high consumption of fish as well as food with whole grains and a low consumption of refined products, sugar and sweets as well as cold cured meat, is connected with a lower risk of the metabolic obesity normal weight syndrome and with a decreased risk of lower HDL cholesterol concentration and increased glucose concentration. The results achieved by us are in conformity with the recommendations related to the diet-dependent diseases prophylactics, advising to increase the fish consumption, replace refined grains with whole grains and limit the consumption of sugar and sweets.

## Abbreviations

BMI: Body mass index
MONW: Metabolic obesity and normal weight
PONS: POlish- Norwegian study
IPAQ: International physical activity questionnaire
MET: Metabolic equivalent of task
FFQ: Food frequency questionnaire
KMO: Kaiser-Meyer-Olkin
MSA: Measure of sampling adequacy
OR: Odds ratio
T: Tertile
DASH: Dietary approaches to stop hypertension
